# Evaluating the impact of a new educational tool on understanding of polygenic risk scores for alcohol use disorder

**DOI:** 10.3389/fpsyt.2022.1025483

**Published:** 2022-11-23

**Authors:** Morgan N. Driver, Sally I-Chun Kuo, Lia Petronio, Deanna Brockman, Jacqueline S. Dron, Jehannine Austin, Danielle M. Dick

**Affiliations:** ^1^Department of Human and Molecular Genetics, Virginia Commonwealth University, Richmond, VA, United States; ^2^Department of Psychiatry, Robert Wood Johnson Medical School, Rutgers University, Piscataway, NJ, United States; ^3^Broad Institute of MIT and Harvard, Cambridge, MA, United States; ^4^Color Health Inc., Burlingame, CA, United States; ^5^Department of Medicine, Center for Genomic Medicine, Massachusetts General Hospital, Boston, MA, United States; ^6^Department of Psychiatry, The University of British Columbia, Vancouver, BC, Canada; ^7^Department of Medical Genetics, The University of British Columbia, Vancouver, BC, Canada; ^8^Rutgers Addiction Research Center, Brain Health Institute, Rutgers University, Piscataway, NJ, United States

**Keywords:** polygenic risk scores, alcohol use disorder, personalized medicine, genetic risk, prevention

## Abstract

**Introduction:**

As gene identification efforts have advanced in psychiatry, so have aspirations to use genome-wide polygenic information for prevention and intervention. Although polygenic risk scores (PRS) for substance use and psychiatric outcomes are not yet available in clinical settings, individuals can access their PRS through online direct-to-consumer resources. One of these widely used websites reports that alcohol use disorder is the third most requested PRS out of >1,000 conditions. However, data indicate that there are misunderstandings about complex genetic concepts, with a lower understanding of PRS being associated with a more negative impact of receiving polygenic risk information. There is a need to develop and evaluate educational tools to increase understanding of PRS.

**Methods:**

We conducted a randomized controlled trial to evaluate the impact of web-based educational information on understanding of PRS for alcohol use disorder. A total of 325 college students (70.4% female; 43.6% White; mean age = 18.9 years) from an urban, diverse university completed the study.

**Results:**

Overall, participants were highly satisfied with the educational information. Results from a one-way ANOVA indicated that there was a significant increase in overall understanding of PRS for alcohol use disorder (*p*-value < 0.001), among individuals who received educational information about PRS and alcohol use disorder, as compared to receiving no accompanying information (adj. *p*-value < 0.001), or educational information about alcohol use disorder only (adj. *p*-value < 0.001).

**Discussion:**

These findings suggest that the web-based educational tool could be provided alongside polygenic risk information in order to enhance understanding and interpretation of the information.

**Clinical trial registration:**

[ClinicalTrials.gov], identifier [NCT05143073].

## Introduction

The basis of precision medicine is to use an individual’s personal genetic information, along with lifestyle information and personal medical history, to make more effective clinical decisions regarding health outcomes (1). For many common complex health outcomes, including psychiatric conditions, an individuals’ genetic liability is calculated using estimates from genome-wide association studies (GWAS), with risk estimates provided in the form of polygenic risk scores (PRS) which sum information about risk-enhancing variants detected across the genome (2).

Providing genetic risk information in the form of a PRS is quite different from genetic feedback that has typically been provided in medicine. Historically, genetic testing has focused on single gene disorders, with results indicating either the presence or absence of a disease-causing variant. These genetic testing results have traditionally been presented in clinical settings by health care professionals such as genetic counselors, who are trained to educate people about the inheritance of genetic conditions and communicate genetic test results (3). However, there are limited numbers of genetic counselors (4) and PRS are most commonly accessed through online direct-to-consumer (DTC) websites, not in a clinical setting (5, 6).

Although PRS are not yet commonly used in clinical settings, they are already available through free, online resources. There has been an exponential increase in the provision of DTC genetic information, with more than 26 million individuals having participated in DTC genetic testing by 2019 (7). Public websites allow individuals to upload raw genetic data obtained from DTC genetic testing companies to compute PRS for a variety of health conditions, including cancer, coronary artery disease, and psychiatric conditions (6). User data from one of these websites illustrates a parallel exponential increase in individuals accessing PRS over the last several years, with the third most frequently accessed PRS being for alcohol dependence (6). Additionally, a recent study found that 80–90% of young adults were interested in receiving their genetic feedback for alcohol use disorder, depression, and anxiety (8).

However, there are concerns about how individuals will understand and interpret PRS for complex health outcomes. PRS represent complex genetic risk information and their interpretation is further complicated by logistical constraints surrounding the calculation of genetic liability in the form of PRS (9, 10). PRS currently only capture a small amount of the variance in a trait which limits their predictive ability (9). Additionally, GWAS are primarily conducted in samples of European ancestry which limits the utility of PRS in diverse populations because PRS based on samples of European ancestries predict poorly in samples of non-European ancestries (9). Many people may not be aware of these issues and limitations when receiving and interpreting PRS information. These complexities may require individuals to have greater genetic and statistical knowledge to understand PRS information as compared to what is needed to understand genetic test results for a single gene disorder.

Indeed, there is evidence of substantial misunderstandings about the contribution of genetic and environmental factors to complex conditions (8, 11, 12), which may impact one’s ability to accurately interpret complex genetic feedback. For example, one study found that 25% of young adults did not know whether substance use and psychiatric conditions were influenced by only one gene or many different genes (8). Results of a recent study showed that 74.4% of the individuals who accessed PRS through a DTC website incorrectly answered at least one of the questions that assessed understanding and interpretation of PRS (13). Concerningly, the individuals who had a lower understanding of PRS had a more negative psychological reaction to receiving their PRS (13).

Given the misunderstandings related to complex genetic concepts and the increasing access to PRS *via* public resources, there is an urgent need to design and evaluate educational materials that can be used to help individuals understand and interpret their PRS information. One of the first efforts in this area has been led by a team of geneticists, clinicians, data visualization specialists, and software developers at the Broad Institute (14). They created a mock PRS report for cardiovascular disease with educational information about PRS, cardiovascular disease, and ways to reduce disease risk. User feedback (*n* = 10) suggested that the use of color, simple graphics, and percentiles helped with PRS interpretation, but the use of static reports were not ideal for disclosure and education of complex genetic results. The team adapted the mock report to create a user-centered, dynamic narrative visualization tool composed of animated graphics aided by simple, repetitive text to help communicate information about PRS and personalized medicine (14). In the present study, we adapted the visualization tool to include additional text and graphics that communicate information about alcohol use disorder since alcohol use disorder is one of the most accessed PRS through DTC resources (6). More importantly, alcohol use disorder is influenced by both genetic factors and environmental factors and there are actionable ways to reduce one’s risk for developing alcohol use disorder through behavior, such as reducing or eliminating alcohol consumption. This uniquely positions alcohol use disorder as a starting phenotype as researchers begin to explore the feasibility and appropriateness of returning PRS for substance use and psychiatric outcomes.

The primary goal of the present study was to adapt the web-based educational tool from Brockman et al. (14) for alcohol use disorder and use a randomized controlled trial to evaluate the impact of the tool on understanding of PRS for alcohol use disorder in a sample of emerging adults. We recruited a sample of college students to participate in the randomized controlled trial because emerging adulthood is a critical period for the onset of problematic alcohol use behavior (15), with college students engaging in high rates of risky drinking behavior and typically using more alcohol than their non-college attending peers (16). The randomized controlled trial consisted of three conditions: (1) A control condition in which participants only received a survey assessment, (2) an intervention condition in which participants received general information about alcohol use disorder, and (3) an intervention condition in which participants received information about PRS and alcohol use disorder. We evaluated the efficacy of the educational information by assessing participants’ understanding of PRS for alcohol use disorder across the three conditions. We hypothesized that receiving information about PRS and alcohol use disorder would result in greater understanding of PRS for alcohol use disorder compared to both the control condition and intervention condition in which participants received information only about alcohol use disorder. Additionally, we assessed satisfaction with the educational information and whether the effect of the intervention varied across participants’ demographic characteristics.

## Materials and methods

### Participants and recruitment

Participants for the study were recruited through Psychology’s SONA (PsychSONA) system at an urban, public university during the fall 2021 semester. PsychSONA is a cloud-based participation and experiment management software that allows researchers to schedule both online and lab studies, recruit participants, monitor participants’ study related activities, and grant study completion credit. Undergraduate students aged 18 years or older had the option of signing up for the study through PsychSONA. The study was described as an hour-long study in which participants could complete an online survey and learn more about alcohol use disorder and genetic risk. Participants were randomly assigned to one of three conditions: (1) A control condition in which participants received only the survey assessment, (2) an intervention condition in which participants received general information about alcohol use disorder, and (3) an intervention condition in which participants received general information about alcohol use disorder and information about PRS. Participants were emailed a unique study link to a REDCap survey which provided additional information about the study and a consent form. Following consent, participants were directed to initial survey items that assessed demographic information, personality, and current alcohol use. Depending on which condition the participants were assigned to, participants were either directed to (1) the remaining survey items, (2) a website that provided educational information about alcohol use disorder and ways to reduce risk, or (3) a website that provided educational information about PRS, alcohol use disorder, and ways to reduce risk. At the bottom of the website, participants were instructed to click a link in order to be redirected back to the survey. The remaining survey items assessed understanding and interpretation of PRS for alcohol use disorder through the use of hypothetical PRS scenarios. Participants within each condition of the randomized controlled trial were randomly assigned to receive the PRS scenarios in one of two different orders: (1) Below-average PRS, average PRS, above-average PRS or (2) above-average PRS, average PRS, below-average PRS. Approximately 50% of the participants in each condition received the scenarios in the first order and 50% received the scenarios in the second order. The additional randomization ensured that there were no order effects associated with the presentation of the various levels of genetic risk in the scenarios. A flow chart of the study design can be visualized in Supplementary Figure 1. After completion of the study, the research coordinator granted one credit to each participant through the PsychSONA system. All data was collected through REDCap (17). All procedures were approved by the University’s Institutional Review Board.

### Educational information

The educational information evaluated in this study was adapted from the coronary artery disease PRS dynamic explainer that was designed and developed by a team of geneticists, clinicians, software developers, and data visualization experts as a collaboration between the Broad Institute’s Cardiovascular Disease Initiative, IBM Research, and Massachusetts General Hospital (14). The original content was developed to help educate users about PRS and ways to reduce risk for cardiovascular disease using both text and visual aids. The website URL for the original PRS dynamic explainer can be found in the footnote^1^. The development of this PRS dynamic explainer was informed by interview feedback on mock PRS reports. Scroll-based techniques were used to introduce concepts in manageable chunks, step-by-step, with corresponding graphics that animate as the user scrolls, allowing users to control the pace of information they receive and create a clear connection between the textual explanation and graphical representation of each concept. Short sentences that use simple wording and repetitive phrases were used throughout the site to enhance comprehension. Color coding was used as the key element to communicate different levels of genetic risk with teal indicating lower genetic risk, gray indicating average genetic risk, and red indicating higher genetic risk (14). These colors were used to highlight key terms throughout the written narrative and to encode the risk information in the corresponding graphics and labels, in order to establish a continuum between the written explanation and visual representation of each concept. The educational information in the present study was adapted from the original site to focus on alcohol use disorder rather than coronary artery disease. See Supplementary Figures 2–5 for more details about the educational information.

#### Intervention condition 1 (AUD Edu)

The information provided to participants in the AUD Edu condition was related to alcohol use disorder, including a definition, prevalence, consequences, risk factors, and risk-reducing strategies. The content was developed based on educational information available through public websites, including the National Institute of Alcohol Abuse and Alcoholism, Mayo Clinic, and the National Survey on Drug Use and Health. Simple graphics were designed to easily communicate risk-reducing strategies, such as measuring drinks, finding alternative activities, and avoiding places that trigger drinking.

#### Intervention condition 2 (PRS + AUD Edu)

The information provided to participants in the PRS + AUD Edu condition explained PRS by discussing genetic variation, risk variants, how PRS are calculated, and how they can be interpreted. The participants also received the same information as the AUD Edu condition that related to alcohol use disorder, including a definition, prevalence, consequences, risk factors, and risk-reducing strategies.

#### Control condition

Participants in the control condition did not receive an educational intervention prior to completing the study.

### Measures

#### Time spent accessing educational information

For participants in the AUD Edu condition and PRS + AUD Edu condition, time spent accessing the website which contained the educational information was calculated using timestamps recorded through REDCap. The first timestamp was recorded when participants were presented with the link to the educational information and the second timestamp was recorded when participants clicked the link at the end of the website containing the educational information and returned to the survey. The time between these two time points was calculated in order to best estimate how long participants spent accessing the educational information presented to them.

#### Satisfaction with the educational materials

Satisfaction was assessed using a series of items directly related to the presentation of information and content provided. Example items included “The animation helped explain concepts,” “I learned new information about alcohol use disorder as part of this program,” and “The pacing of the information was manageable.” These items were rated on a five-point scale from strongly disagree to strongly agree. Participants were also asked to rate how useful and satisfied they were with the different sections of the educational material on a five-point scale.

#### Understanding of polygenic risk scores

The items used to assess understanding of PRS for alcohol use disorder were adapted from items used in Peck et al. (13). Two items were used to assess general understanding of PRS with response options of “true,” “false,” and “don’t know.” An example item was “A genetic risk score includes only some of the genetic factors that can contribute to risk for the condition.” Additionally, 12 items were used to assess understanding of different levels of PRS for alcohol use disorder. Participants were asked to imagine that they received each PRS for alcohol use disorder (below-average, average, and above-average) and respond to a series of questions. Below-average risk was indicated using a graph in which the PRS was in the 30th percentile, average risk was indicated using a graph in which the PRS was in the 50th percentile, and above-average risk was indicated using a graph in which the PRS was in the 75th percentile. See Supplementary Figure 6 for additional details regarding the display of hypothetical PRS. Example items for the below-average PRS scenario included “You will definitely develop alcohol use disorder” and “You have a lower chance than the average person to develop alcohol use disorder.” Response options included “agree,” “disagree,” and “don’t know.” The 14 items were scored as either correct or incorrect, with “don’t know” also being scored as incorrect, to create an overall sum score (range 0–14). Separate scores were calculated for understanding of each hypothetical PRS (range 0–4).

#### Drinking status

Drinking status was measured using the frequency item from the Alcohol Use Disorder Identification Test (AUDIT) (18). A total of 34.8% of the full sample “never” used alcohol, 27.4% used alcohol “monthly or less,” 23.4% used alcohol “2 to 4 times a month,” 10.5% used alcohol “2 to 3 times a week,” 1.5% used alcohol “4 or more times a week,” and 2.5% chose not to answer. In view of the distribution, participants who responded “never” to the alcohol frequency item were coded as 0 to indicate that they had not previously used alcohol and participants who responded at least “monthly or less” were coded as 1 to indicate that they had previously used alcohol.

#### Personal history of alcohol problems

Personal history of having an alcohol use disorder was assessed using the question “Have you ever been diagnosed with an alcohol use disorder by a healthcare professional?” Response options were “yes,” “no,” and “don’t know.”

#### Family history of alcohol problems

Participants answered separate questions about alcohol problems for four types of biological relatives: Mother, father, aunts/uncles/grandparents, and siblings (19). An example question was: “Do you think your biological mother has ever had a drinking problem? (By drinking problem we mean that her drinking caused problems at home, at work, with her health, or with the police, or that she received alcohol treatment.).” The questions were repeated for each type of relative. Response options for each question were “yes,” “no,” and “I don’t know.” Family history items related to alcohol problems were combined into an overall binary variable to indicate whether or not the participant had any first- or second-degree relatives with a history of alcohol problems.

#### Personality

Six items from the Big Five Inventory (BFI) (20) were included to assess extraversion and neuroticism. Three items from the short UPPS-P Impulsive Behavior Scale (SUPPS-P) (21) were included to assess sensation seeking. Items were coded according to the scoring guidelines of the BFI and SUPPS-P and averaged to create an overall score for each personality dimension (extraversion, neuroticism, and sensation seeking).

#### Demographic variables

Sex assigned at birth, race/ethnicity, and age were the primary demographic variables included in the analyses. A detailed breakdown of race/ethnicity is reported in Table 1. Because approximately half of the sample self-identified as White (43.6%), race/ethnicity was coded as a binary variable. Individuals who self-identified as White were coded as 0, and individuals who self-identified as Asian, Black/African American, Hispanic/Latino, American Indian/Alaska Native, Native Hawaiian/Pacific Islander, or more than one race were coded as 1.

**TABLE 1:** Demographic characteristics for participants in each condition of the randomized controlled trial and for the total sample.

Demographic characteristic	Control	AUD Edu	PRS + AUD	Total	*F*/*X*^2^
	(*n* = 109)	(*n* = 105)	Edu (*n* = 111)	(*n* = 325)	*(P*-value)
Age					0.38 (0.69)
Mean (SD)	18.85 (1.25)	19.06 (2.66)	18.89 (1.25)	18.93 (1.82)	
Sex assigned at birth, *n* (%)					5.43 (0.07)
Male	41 (37.6)	29 (27.6)	26 (23.6)	96 (29.6)	
Female	68 (62.4)	76 (72.4)	84 (76.4)	228 (70.4)	
Race/Ethnicity, *n* (%)					7.50 (0.82)
American Indian/Alaska native	0 (0.0)	1 (1.0)	0 (0.0)	1 (0.3)	
Asian	20 (18.5)	21 (20.2)	19 (17.4)	60 (18.7)	
Black/African American	24 (22.2)	24 (23.1)	30 (27.5)	78 (24.3)	
Hispanic/Latino	4 (3.7)	7 (6.7)	8 (7.3)	19 (5.9)	
More than one race	8 (7.4)	7 (6.7)	7 (6.4)	22 (6.9)	
Native Hawaiian/Other Pacific Islander	1 (0.9)	0 (0.0)	0 (0.0)	1 (0.3)	
White	51 (47.2)	44 (42.3)	45 (41.3)	140 (43.6)	
Unknown	0 (0.0)	0 (0.0)	0 (0.0)	0 (0.0)	
Race/Ethnicity (Binary), *n* (%)					0.88 (0.64)
White	51 (47.2)	44 (42.3)	45 (41.3)	140 (43.6)	
Racial/Ethnic minority	57 (52.8)	60 (57.7)	64 (58.7)	181 (56.4)	
Previous AUD Diagnosis, *n* (%)					4.07 (0.40)
Yes	0 (0.0)	2 (1.9)	1 (0.9)	3 (0.9)	
No	109 (100.0)	102 (98.1)	109 (98.2)	320 (98.8)	
Don’t know	0 (0.0)	0 (0.0)	1 (0.9)	1 (0.3)	
Alcohol use, *n* (%)					6.04 (0.05)
Yes	59 (55.7)	67 (65.7)	78 (71.6)	204 (64.4)	
No	47 (44.3)	35 (34.3)	31 (28.4)	113 (35.7)	
Family history of alcohol problems, *n* (%)					0.40 (0.82)
Yes	53 (48.6)	55 (52.9)	55 (50.0)	163 (50.5)	
No	56 (51.4)	49 (47.1)	55 (50.0)	160 (49.5)	
Extraversion					1.34 (0.26)
Mean (SD)	2.05 (1.02)	2.08 (1.13)	2.27 (1.12)	2.13 (1.09)	
Neuroticism					0.02 (0.98)
Mean (SD)	2.22 (1.01)	2.24 (0.86)	2.25 (0.98)	2.24 (0.95)	
Sensation seeking					0.69 (0.51)
Mean (SD)	1.73 (0.77)	1.83 (0.65)	1.83 (0.68)	1.8 (0.70)	

Bonferroni corrected significance threshold *p* < 0.05/10 = 0.005. Statistical information presented in the last column of the table assess differences across the three study conditions.

### Analyses

Descriptive analyses were used to describe demographic information for each condition and for the full sample. ANOVA methods and chi-squared tests were used to ensure that randomization was successful across the three conditions of the randomized controlled trial. Counts and frequencies were used to examine satisfaction items presented to participants in the AUD Edu condition and PRS + AUD Edu condition. Counts and frequencies were also used to investigate individual items that assessed understanding and interpretation of PRS. To examine the effectiveness of the educational information, a one-way ANOVA was used to compare mean understanding of PRS for alcohol use disorder between the control condition, AUD Edu condition, and PRS + AUD Edu condition. *Post-hoc* tests were conducted to examine where the differences occurred. To examine moderators influencing the effects of the educational information, two-way ANOVAs were conducted to examine interactions between the interventions and demographic characteristics (i.e., sex, race/ethnicity, drinking status, and family history of alcohol problems) on understanding of PRS for alcohol use disorder. Additionally, a linear regression model was conducted to assess the robustness of the effect of the intervention on overall understanding of PRS for alcohol use disorder while controlling for demographic characteristics (e.g., sex, age, and race/ethnicity) and characteristics that influence individual risk for alcohol use disorder (e.g., family history of alcohol problems, drinking status, and personality). Only three participants in the sample indicated a previous diagnosis of alcohol use disorder so this variable was not included in the model. Two variables were included to indicate whether or not participants received educational information about alcohol use disorder and received educational information about PRS. In order to assess whether understanding varies across levels of genetic risk, one-way ANOVAs were used to compare whether there were mean differences in the understanding of below-average, average, and above-average PRS within each condition. All analyses were conducted using R 4.0.4 software (22). The analytic plan and hypotheses were pre-registered through the Open Science Framework (osf.io/efh6j). The study was also registered on ClinicalTrials.gov (Identifier: NCT05143073).

## Results

### Sample characteristics

**FIGURE 1:**
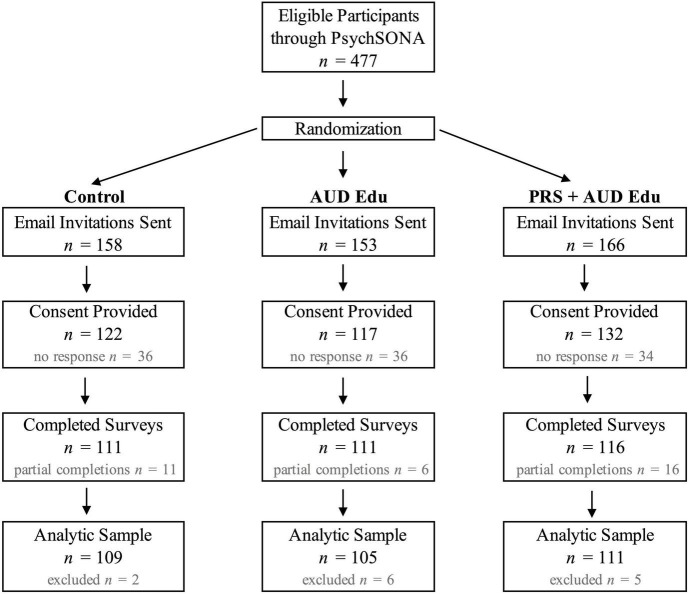
Consort diagram illustrating recruitment details for the study.

Figure 1 displays the recruitment details for each condition of the randomized controlled trial. A total of 477 participants signed up to participate in the study and were randomly assigned to a study condition, 371 participants (77.8%) consented to participate in the study, and 338 participants (70.9%) completed the study. In total, 12 participants were removed from analyses due to high response rates of “I choose not to answer” (>25% of all survey items). One participant completed the study twice, and the participant’s second survey completion was removed from the analyses.

The final analytic sample included 325 participants: 109 participants in the control condition, 105 participants in the AUD Edu condition, and 111 participants in the PRS + AUD Edu condition. Participant demographic characteristics for each condition of the randomized controlled trial, as well as characteristics of the entire study sample, are displayed in Table 1. A total of 70.4% of the sample self-reported sex assigned at birth as female. A total of 43.6% of the sample self-identified as White, 24.3% of the sample self-identified as Black/African American, and 18.7% of the sample self-identified as Asian. The demographic characteristics of the full sample are generally reflective of overall university demographics, with a greater percentage of females (62%) than males (38%) and 45.8% of the undergraduate student population self-identifying as White, 17.5% as Black/African American, and 13.3% as Asian. The mean age of the sample was 18.93 years. Using a series of comparison tests with a Bonferroni corrected *p*-value threshold of 0.005, there were no significant differences in demographic characteristics across the three conditions, indicating that randomization of participants was effective.

### Satisfaction with educational websites

On average, participants in the AUD Edu condition spent 0.87 min (SD = 0.8) accessing the educational information regarding alcohol use disorder and participants in the PRS + AUD Edu condition spent 1.77 min (SD = 1.4) accessing the educational information regarding PRS and alcohol use disorder. Overall, participants in both the AUD Edu condition and the PRS + AUD Edu condition appeared to be satisfied with different aspects of the educational websites, including length, order of the content, pacing, and online delivery method. A total of 73 participants (69.5%) in the AUD Edu condition and 87 participants (78.4%) in the PRS + AUD Edu condition agreed or strongly agreed that they enjoyed the website. Additionally, almost all of the participants in the PRS + AUD Edu condition (96.4%) reported that they learned new information about complex genetic risk through the website. Over 90% of participants in the PRS + AUD Edu condition thought that the way in which the genetic risk information was displayed, including the animation and color choice, was helpful.

### Responses to items that assess understanding of polygenic risk scores

Descriptive analyses were used to investigate responses to the individual items which assessed understanding and interpretation of PRS for alcohol use disorder. Counts and frequencies of response options for each item are displayed in Table 2. Strikingly, participants across the three conditions had the highest incorrect response rate to the item “you have a chance of over X% to develop alcohol use disorder” in each PRS scenario. On average approximately 30% of participants in the PRS + AUD Edu condition correctly responded to these statements, while approximately 15% of participants in both the control condition and AUD Edu condition correctly responded to these statements. Interestingly, there was a discrepancy in understanding one’s own risk compared to understanding one’s risk as it relates to others in the population. For example, in the control condition, 93 participants (85.3%) understood that they had a higher chance than the average person to develop alcohol use disorder when provided with the above-average genetic risk score, but 84 participants (77.1%) incorrectly agreed that their chance of developing alcohol use disorder was over 75% when provided with a PRS that was greater than the 75th percentile. This pattern occurred across the three conditions, as well as for each level of PRS provided.

**TABLE 2:** Counts and frequencies of response options for each item that assessed understanding of polygenic risk scores for alcohol use disorder.

Item	Correct response	Responses	Control		AUD Edu		PRS + AUD Edu	
					
			*n*	%	*n*	%	*n*	%
**A genetic risk score:**								
Includes only some of the genetic factors that can contribute to risk for the condition	True	**True**	**48**	**44.0**	**66**	**62.9**	**69**	**62.2**
		False	14	15.6	9	8.6	17	15.3
		Don’t know	44	40.4	30	28.6	25	22.5
Shows that your lifestyle and environment play no role in whether you develop the condition	False	True	13	11.9	13	12.4	12	10.8
		**False**	**60**	**55.1**	**65**	**61.9**	**86**	**77.5**
		Don’t know	36	33.0	27	25.7	13	11.7
**Below-average genetic risk score:**								
You have a lower chance than the average person to develop alcohol use disorder	Agree	**Agree**	**94**	**87.0**	**89**	**84.8**	**100**	**90.1**
		Disagree	5	4.6	8	7.6	6	5.4
		Don’t know	9	8.3	8	7.6	5	4.5
You will definitely develop alcohol use disorder	Disagree	Agree	1	0.9	2	1.9	3	2.7
		**Disagree**	**88**	**81.5**	**90**	**85.7**	**100**	**90.1**
		Don’t know	19	17.6	13	12.4	8	7.2
You have a chance of just over 30% to develop alcohol use disorder	Disagree	Agree	79	73.2	84	80.0	70	63.1
		**Disagree**	**15**	**13.9**	**12**	**11.4**	**33**	**29.7**
		Don’t know	14	13.0	9	8.6	8	7.2
You will definitely NOT develop alcohol use disorder	Disagree	Agree	15	14.0	16	15.2	11	9.9
		**Disagree**	**70**	**65.4**	**66**	**62.7**	**80**	**72.1**
		Don’t know	22	20.6	23	21.9	20	18.0
**Average genetic risk score:**								
You have a similar chance as the average person to develop alcohol use disorder	Agree	**Agree**	**77**	**70.6**	**77**	**74.0**	**89**	**80.2**
		Disagree	16	14.7	17	16.4	14	12.6
		Don’t know	16	14.7	10	9.6	8	7.2
You will definitely develop alcohol use disorder	Disagree	Agree	18	16.7	13	12.4	10	9.0
		**Disagree**	**67**	**62.0**	**72**	**68.6**	**93**	**83.8**
		Don’t know	23	21.3	20	12.4	8	7.2
You have a chance of just over 50% to develop alcohol use disorder	Disagree	Agree	75	68.8	80	76.2	69	62.2
		**Disagree**	**19**	**17.4**	**17**	**16.2**	**33**	**29.7**
		Don’t know	15	13.8	8	7.6	9	8.1
You will definitely NOT develop alcohol use disorder	Disagree	Agree	6	5.5	10	9.5	2	1.8
		**Disagree**	**73**	**67.0**	**72**	**68.6**	**94**	**84.7**
		Don’t know	30	27.5	23	21.9	15	13.5
**Above-average genetic risk score:**								
You have a higher chance than the average person to develop alcohol use disorder	Agree	**Agree**	**93**	**85.3**	**87**	**83.7**	**97**	**87.4**
		Disagree	8	7.3	13	12.5	8	7.2
		Don’t know	8	7.3	4	3.9	6	5.4
You will definitely develop alcohol use disorder	Disagree	Agree	34	31.5	29	27.9	29	26.1
		**Disagree**	**54**	**50.0**	**70**	**67.3**	**75**	**67.6**
		Don’t know	20	18.5	5	4.8	7	6.3
You have a chance of just over 75% to develop alcohol use disorder	Disagree	Agree	84	77.1	83	79.8	73	66.4
		**Disagree**	**15**	**13.8**	**16**	**15.4**	**31**	**28.2**
		Don’t know	10	9.2	5	4.8	6	5.5
You will definitely NOT develop alcohol use disorder	Disagree	Agree	8	7.3	14	13.5	9	8.1
		**Disagree**	**81**	**74.3**	**78**	**75.0**	**93**	**83.8**
		Don’t know	20	18.4	12	11.5	9	8.1

Boldface text indicates the correct response to the item, as well as the *n* (%) of that correct response in each of the three conditions.

### Overall understanding of polygenic risk scores for alcohol use disorder

There was a significant difference in overall understanding of PRS between the three conditions (*p*-value < 0.001). The mean score for the control condition was 7.86 (SD = 3.06), the mean score for the AUD Edu condition was 8.38 (SD = 2.62), and the mean score for the PRS + AUD Edu condition was 9.67 (SD = 2.79), where higher scores indicated a greater understanding of hypothetical PRS for alcohol use disorder. Significant mean differences occurred between the control condition and PRS + AUD Edu condition (diff = 1.81; adj. *p*-value < 0.001) and between the AUD Edu condition and the PRS + AUD Edu condition (diff = 1.29; adj. *p*-value < 0.005). There was not a significant difference in understanding of PRS between the control condition and the AUD Edu condition (diff = 0.52; adj. *p*-value = 0.37). These results are summarized in Table 3.

**TABLE 3:** Mean (SD) understanding of polygenic risk scores for alcohol use disorder across the three conditions of the randomized controlled trial.

Condition	Mean (SD)	*F*-test
Control	7.86 (3.06)^a^	*F* (2, 322) = 11.96; *p* < 0.0001
AUD Edu	8.38 (2.62)^b^	
PRS + AUD Edu	9.67 (2.79)^a,b^	

Values that share a superscript are significantly different (adj. *p* < 0.01). ^a^Refers to significant differences between the control condition and the PRS + AUD Edu condition. ^b^Refers to significant differences between the AUD Edu condition and PRS + AUD Edu condition. Fourteen items were used to assess understanding of hypothetical polygenic risk scores for alcohol use disorder. Items were scored as correct or incorrect and summed to create an overall sum score. Range = 0–14.

There were no significant interactions between the intervention condition and demographic characteristics (i.e., sex, race/ethnicity, drinking status, and family history of alcohol problems) on understanding of PRS for alcohol use disorder (Table 4). The effect of the intervention was consistent across females and males, individuals who self-identified as White and individuals who self-identified as other races/ethnicities, individuals who use and do not use alcohol, and individuals with and without a family history of alcohol problems. Additional exploratory analyses demonstrated no significant interactions between the intervention condition and race/ethnicity when categorized as White, Black/African American, and Asian.

**TABLE 4:** Results of two-way ANOVAs examining interactions between the study condition and demographic characteristics.

Moderator	Binary category	Control	AUD Edu	PRS + AUD Edu	Interaction		
		M (SD)	M (SD)	M (SD)	*F*-value	*df*	*P*-value
Sex	Male	7.97 (3.26)	8.14 (2.92)	10.60 (2.73)	1.51	2	0.22
	Female	7.79 (2.96)	8.47 (2.51)	9.40 (2.78)			
Race/Ethnicity	White	8.85 (2.62)	8.82 (2.70)	10.10 (3.14)	1.34	2	0.26
	Non-white	7.05 (3.16)	8.06 (2.56)	9.39 (2.55)			
Drinking status	Drinker	8.14 (2.94)	8.51 (2.33)	9.56 (3.01)	1.12	2	0.33
	Non-drinker	7.40 (3.20)	8.40 (3.02)	10.00 (2.23)			
Family history of alcohol problems	No family history	7.59 (3.26)	8.63 (2.92)	9.79 (2.81)	0.94	2	0.40
	Family history	8.14 (2.83)	8.22 (2.32)	9.49 (2.78)			

Results from four separate two-way ANOVAs are displayed in this table.

Additionally, the effect of receiving information about PRS on understanding appears to be robust while controlling for demographic characteristics and characteristics generally associated with substance use behaviors (e.g., family history of alcohol problems, drinking status, and personality). Receiving educational information about PRS was significantly associated with understanding of PRS for alcohol use disorder (*p* < 0.01) while controlling for individual characteristics (Supplementary Table 1).

### Understanding of different levels of polygenic risk scores

Lastly, we assessed whether understanding of PRS varies across different levels of genetic risk. Table 5 displays the mean understanding scores for each level of PRS (below-average, average, and above-average) for alcohol use disorder in each condition. Participants in the AUD Edu condition and PRS + AUD Edu condition had a similar understanding of each PRS regardless of whether the PRS was below-average, average, or above-average. There was a significant difference in understanding of the different PRS in the control condition (*p*-value < 0.01), with *post-hoc* analyses demonstrating a significant mean difference between understanding of below-average PRS for alcohol use disorder and understanding of average PRS for alcohol use disorder (diff = −0.32; adj. *p*-value < 0.05). The mean difference between understanding of a below-average PRS and an above-average PRS was −0.25 (adj. *p*-value = 0.08). This suggests that participants in the control condition had a slightly better understanding of below-average PRS compared to average or above-average PRS.

**TABLE 5:** Mean (SD) understanding of each level of hypothetical polygenic risk score for alcohol use disorder.

Condition	Below-average PRS	Average PRS	Above-average PRS	*F*-test
	M (SD)	M (SD)	M (SD)	
Control	2.48 (1.04)	2.16 (1.20)	2.23 (1.02)	*F* (1.9,201.4) = 5.09; *p* < 0.01
AUD Edu	2.45 (0.93)	2.27 (0.99)	2.41 (0.80)	*F* (2,206) = 2.55; *p* = 0.08
PRS + AUD Edu	2.82 (0.96)	2.78 (0.99)	2.67 (0.95)	*F* (2,220) = 2.22; *p* = 0.11

Four items were included to assess understanding of each PRS. Items were scored as correct or incorrect and summed to create a sum score for each scenario. Range = 0–4. PRS, polygenic risk score.

## Discussion

This is the first randomized controlled trial designed to evaluate the efficacy of educational information delivered through a web-based educational tool intended to increase understanding and interpretation of PRS for alcohol use disorder. The randomized controlled trial consisted of a control condition in which participants only received a survey assessment, an intervention condition in which participants received educational information about alcohol use disorder, and an intervention condition in which participants received information about PRS and alcohol use disorder. The educational information which explained PRS focused on genetic variation, risk variants, how PRS are created, and how they can be interpreted. Results showed that the educational information about PRS and alcohol use disorder significantly increased participants’ ability to understand and interpret hypothetical PRS for alcohol use disorder. The effect of receiving educational information about PRS was consistent across several demographic characteristics, including sex and race/ethnicity, demonstrating that the intervention has the potential to be equally effective across individuals of diverse backgrounds.

Additionally, participants in the PRS + AUD Edu condition had a similar understanding of each PRS regardless of whether the PRS was below-average, average, or above-average, suggesting that the intervention increased general understanding of PRS. However, participants in the control condition had a slightly better understanding of below-average PRS compared to average or above-average PRS. This could further exacerbate a negative impact of receiving an above-average PRS, as a lower understanding of PRS has been previously shown to be associated with more negative psychosocial reactions (13). These findings further demonstrate the importance of providing individuals with educational information about PRS prior to receiving genetic risk information.

It is important to note that although understanding of PRS did increase significantly, participants on average answered approximately 10 out of 14 items correct (69.1%). This suggests that there are still ways that the educational information and provision of PRS could be improved. Response rates for the individual items that assessed understanding and interpretation of PRS revealed that a majority of participants understood how the PRS impacted their chance of developing alcohol use disorder as compared to others but less than 30% of participants understood how the PRS related to their overall chance of developing alcohol use disorder. Presenting PRS as percentiles may have confused participants, as a majority of participants seemed to believe that the percentile reflected their chance of developing alcohol use disorder. Our data support existing evidence (23, 24) that providing absolute risk information to participants may aid in their understanding and interpretation of PRS. At the time of this study, translating PRS into absolute risk was complex, and not yet being routinely performed so we focused on ways to improve the comprehensibility of available PRS information. Currently, new tools have been developed that can convert PRS into absolute risk (25), and future studies could assess the outcomes of using these strategies in risk communication interventions. Further, it is possible that the limited amount of variance currently accounted for by PRS (9), and limitations in portability of PRS across ancestral groups (9) may be contributing to confusion about how PRS relate to one’s risk of developing problems.

Promisingly, participants assigned to the intervention conditions were highly satisfied with the educational information. Participants liked many aspects of the website, including the animation, colors, and presentation of the information. In the future, the content can be adapted to discuss genetic risk for many different complex disorders and diseases, which can be easily implemented as the PRS dynamic explainer is currently an online, publicly accessible website. Additionally, providing educational information about PRS through an effective online website can be a cost-effective strategy for education and can broaden access to education. As demand is high for genetic counseling services, an effective educational resource that can accompany the provision of PRS could be well-utilized and allow genetic counselors to operate to the top of their scope of practice focusing on counseling rather than providing information.

These findings should be interpreted in light of several limitations. First, the randomized controlled trial was conducted in a sample of college students. Although the educational information regarding PRS effectively increased understanding and interpretation of PRS for alcohol use disorder in this sample, the results may not be generalizable to other populations. Replicating these findings in diverse samples is important. Efficacy of the PRS dynamic explainer should be assessed in unaffected populations with diverse educational backgrounds and ages, a sample of clinicians, and affected patient populations. Second, the sample disproportionately consisted of female participants (comprising 70%); accordingly, we did not have power to test for potential sex differences. Our results may be more representative for females. Future studies should aim for equal representation of males and females. Third, the PRS were presented as hypothetical scenarios and may not reflect how an individual would understand and interpret their true PRS for alcohol use disorder. Additional research should be conducted to assess understanding of one’s true PRS information for a variety of different disorders. Additionally, the time participants spent accessing the website was estimated using two timestamps recorded with the survey software; however, we cannot assume that participants used that time to engage with the website. Due to the online nature of this study, there is no way to guarantee that participants read through all of the educational information provided to them or to assess level of engagement with the website.

## Conclusion

In conclusion, the educational information utilized in this randomized controlled trial effectively increased understanding of PRS for alcohol use disorder in a sample of emerging adults. As the possibility of providing PRS information to inform prevention programing, screening, and treatment increases, the need to educate individuals about complex genetic concepts increases as well. Findings of the present study suggest that the PRS dynamic explainer could be used alongside the return of polygenic risk information in order to enhance understanding and interpretation of the genetic risk information. Future research should focus on assessing the effectiveness of the educational information in diverse samples across different age groups and educational background and assess how the information may impact one’s understanding of their true genetic risk information.

## Data availability statement

The raw data supporting the conclusions of this article will be made available by the authors, without undue reservation.

## Ethics statement

The studies involving human participants were reviewed and approved by the Virginia Commonwealth University Institutional Review Board. The patients/participants provided their informed consent to participate in this study through REDCap.

## Author contributions

MD, SK, JA, and DD contributed to conception and design of the study. MD collected data for the study, performed the statistical analysis, and wrote the first draft of the manuscript. LP, DB, and JD created the educational tool used in the study. LP created graphics and adapted versions of the educational tool for use in the study. All authors contributed to manuscript revision, read, and approved the submitted version.

## Footnotes

1
http://polygenicscores.org/explained/

## References

[1] Collins FS Varmus H. A new initiative on precision medicine. *N Engl J Med.* (2015) 372:793–5. 10.1056/NEJMp1500523 25635347 PMC5101938

[2] Visscher PM Wray NR Zhang Q Sklar P McCarthy MI Brown MA 10 years of GWAS discovery: biology, function, and translation. *Am J Hum Genet.* (2017) 101:5–22. 10.1016/j.ajhg.2017.06.005 28686856 PMC5501872

[3] National Society of Genetic Counselors’ Definition Task Force, Resta R Biesecker BB Bennett RL Blum S Hahn SE A new definition of genetic counseling: National Society of Genetic Counselors’ Task Force report. *J Genet Couns.* (2006) 15:77–83. 10.1007/s10897-005-9014-3 16761103

[4] Hoskovec JM Bennett RL Carey ME DaVanzo JE Dougherty M Hahn SE Projecting the supply and demand for certified genetic counselors: a workforce study. *J Genet Couns.* (2018) 27:16–20. 10.1007/s10897-017-0158-8 29052810

[5] Polygenic Risk Score Task Force of the International Common Disease Alliance. Responsible use of polygenic risk scores in the clinic: potential benefits, risks and gaps. *Nat Med.* (2021) 27:1876–84. 10.1038/s41591-021-01549-6 34782789

[6] Folkersen L Pain O Ingason A Werge T Lewis CM Austin J. Impute.me: an open-source, non-profit tool for using data from direct-to-consumer genetic testing to calculate and interpret polygenic risk scores. *Front Genet.* (2020) 11:578. 10.3389/fgene.2020.00578 32714365 PMC7340159

[7] Regalado A. More than 26 million people have taken an at-home ancestry test. *MIT Technol Rev.* (2019). Available online at: https://www.technologyreview.com/2019/02/11/103446/more-than-26-million-people-have-taken-an-at-home-ancestry-test/ (accessed Nov 6, 2022).

[8] Driver MN Kuo SIC Dick DM On Behalf Of The Spit For Science Working Group. Interest in genetic feedback for alcohol use disorder and related substance use and psychiatric outcomes among young adults. *Brain Sci.* (2020) 10:1007. 10.3390/brainsci10121007 33352962 PMC7766419

[9] Lewis ACF Green RC. Polygenic risk scores in the clinic: new perspectives needed on familiar ethical issues. *Genome Med.* (2021) 13:14. 10.1186/s13073-021-00829-7 33509269 PMC7844961

[10] Palk AC Dalvie S de Vries J Martin AR Stein DJ. Potential use of clinical polygenic risk scores in psychiatry – Ethical implications and communicating high polygenic risk. *Philos Ethics Humanit Med.* (2019) 14:4. 10.1186/s13010-019-0073-8 30813945 PMC6391805

[11] Molster C Charles T Samanek A O’Leary P. Australian study on public knowledge of human genetics and health. *PHG.* (2009) 12:84–91. 10.1159/000164684 19039252

[12] Chapman R Likhanov M Selita F Zakharov I Smith-Woolley E Kovas Y. New literacy challenge for the twenty-first century: genetic knowledge is poor even among well educated. *J Community Genet.* (2019) 10:73–84. 10.1007/s12687-018-0363-7 29589204 PMC6325037

[13] Peck L Borle K Folkersen L Austin J. Why do people seek out polygenic risk scores for complex disorders, and how do they understand and react to results? *Eur J Hum Genet.* (2022) 30:81–7. 10.1038/s41431-021-00929-3 34276054 PMC8738734

[14] Brockman DG Petronio L Dron JS Kwon BC Vosburg T Nip L Design and user experience testing of a polygenic score report: a qualitative study of prospective users. *BMC Med Genomics.* (2021) 14:238. 10.1186/s12920-021-01056-0 34598685 PMC8485114

[15] Kessler RC Berglund P Demler O Jin R Merikangas KR Walters EE. Lifetime prevalence and age-of-onset distributions of DSM-IV disorders in the National Comorbidity Survey Replication. *Arch Gen Psychiatry.* (2005) 62:593–602. 10.1001/archpsyc.62.6.593 15939837

[16] Schulenberg JE Johnston LD O’Malley PM Bachman JG Miech RA Patrick ME. *Monitoring the Future National Survey Results on Drug Use, 1975-2017. Volume II, College Students & Adults Ages 19-55. Institute for Social Research.* (2018). Available online at: https://eric.ed.gov/?id=ED589764 (accessed Nov 13, 2020). 10.3998/2027.42/146531

[17] Harris PA Taylor R Thielke R Payne J Gonzalez N Conde JG. Research electronic data capture (REDCap)–a metadata-driven methodology and workflow process for providing translational research informatics support. *J Biomed Inform.* (2009) 42:377–81. 10.1016/j.jbi.2008.08.010 18929686 PMC2700030

[18] Bohn MJ Babor TF Kranzler HR. The Alcohol Use Disorders Identification Test (AUDIT): validation of a screening instrument for use in medical settings. *J Stud Alcohol.* (1995) 56:423–32. 10.15288/jsa.1995.56.423 7674678

[19] Dick DM Nasim A Edwards AC Salvatore JE Cho SB Adkins A Spit for science: launching a longitudinal study of genetic and environmental influences on substance use and emotional health at a large US university. *Front Genet.* (2014) 5:47. 10.3389/fgene.2014.00047 24639683 PMC3944794

[20] John OP Srivastava S. The big five trait taxonomy: history, measurement, and theoretical perspectives. 2nd ed. In: Pervin LA John OP editors. *Handbook of Personality: Theory and Research.* New York, NY: Guilford Press (1999). p. 102–38.

[21] Lynam D Smith G Whiteside S Cyders M. *The UPPS-P: Assessing Five Personality Pathways to Impulsive Behavior (Technical Report).* West Lafayette, IND: Purdue University (2006).

[22] R Core Team. *R: A Language and Environment for Statistical Computing.* Vienna: R Foundation for Statistical Computing (2018).

[23] Zipkin DA Umscheid CA Keating NL Allen E Aung K Beyth R Evidence-based risk communication: a systematic review. *Ann Intern Med.* (2014) 161:270–80. 10.7326/M14-0295 25133362

[24] Gigerenzer G Gaissmaier W Kurz-Milcke E Schwartz LM Woloshin S. Helping doctors and patients make sense of health statistics. *Psychol Sci Public Interest.* (2007) 8:53–96. 26161749 10.1111/j.1539-6053.2008.00033.x

[25] Pain O Gillett AC Austin JC Folkersen L Lewis CM. A tool for translating polygenic scores onto the absolute scale using summary statistics. *Eur J Hum Genet.* (2022) 30:339–48. 10.1038/s41431-021-01028-z 34983942 PMC8904577

