# Supplementary material for: Evaluating the impact of a new educational tool on understanding of polygenic risk scores for alcohol use disorder
Source: Front Psychiatry. 2022 Nov 23;13:1025483. doi: 10.3389/fpsyt.2022.1025483 (PMC9726708; doi:10.3389/fpsyt.2022.1025483)
Supplement: Supplementary file 1 [file Data_Sheet_1.DOCX]

Supplementary Material

# Educational Information

The educational tool used in the present study includes information and materials adapted from the polygenic risk score dynamic explainer (<http://polygenicscores.org/explained/>) to focus on alcohol use disorder. The site used in the study included a disclaimer that specified that the website was being used for research purposes only. Simple text, short sentences, and repetitive phrases were used throughout the site to enhance comprehension (demonstrated in Supplementary Figure 2). Color coding was used to enhance understanding with teal indicating lower genetic risk, gray indicating average genetic risk, and red indicating higher genetic risk (demonstrated in Supplementary Figure 3). The information about alcohol use disorder provided through the website is shown in Supplementary Figure 4. Risk reducing strategies were also presented to participants using a series of simple graphics and short text (examples shown in Supplementary Figure 5). Screenshots of the educational information are included with consent from the developers of the polygenic risk score dynamic explainer.

# List of References for Alcohol Use Disorder Educational Information

Hannah Ritchie and Max Roser (2018) - "Alcohol Consumption". Published online at [OurWorldInData.org](https://ourworldindata.org/). Retrieved from: <https://ourworldindata.org/alcohol-consumption> [Online Resource]

Esser MB, Sherk A, Liu Y, et al. Deaths and Years of Potential Life Lost From Excessive Alcohol Use — United States, 2011–2015. MMWR Morb Mortal Wkly Rep 2020;69:1428–1433. DOI: <http://dx.doi.org/10.15585/mmwr.mm6939a6>

Mayo Clinic Staff. (n.d.). Alcohol use disorder—Symptoms and causes. Mayo Clinic. Retrieved July 19, 2021, from <https://www.mayoclinic.org/diseases-conditions/alcohol-use-disorder/symptoms-causes/syc-20369243>

Understanding Alcohol Use Disorder. (2021, April). National Institute on Alcohol Abuse and Alcoholism (NIAAA). <https://www.niaaa.nih.gov/publications/brochures-and-fact-sheets/understanding-alcohol-use-disorder>

# Hypothetical Polygenic Risk Scores for Alcohol Use Disorder

Participants were provided with three hypothetical polygenic risk scores for alcohol use disorder. The information was presented using a normal distribution with a line that designated the percentile of the polygenic risk score. The graphs were created in a way that models the way in which impute.me, a direct-to-consumer resource, returns polygenic risk scores to consumers (Folkersen et al., 2020), as well as incorporates the use of percentiles similar to the educational information (Brockman, et al. 2021). Below-average risk was indicated using a graph in which the polygenic risk score was above the 30th percentile, average risk was indicated using a graph in which the polygenic risk score was in the 50th percentile, and above-average risk was indicated using a graph in which the polygenic risk score was in the 75th percentile. The graphs used to present this information to the participants are displayed in Supplemental Figure 6.

# Supplementary Figures and Tables

## Supplementary Figures
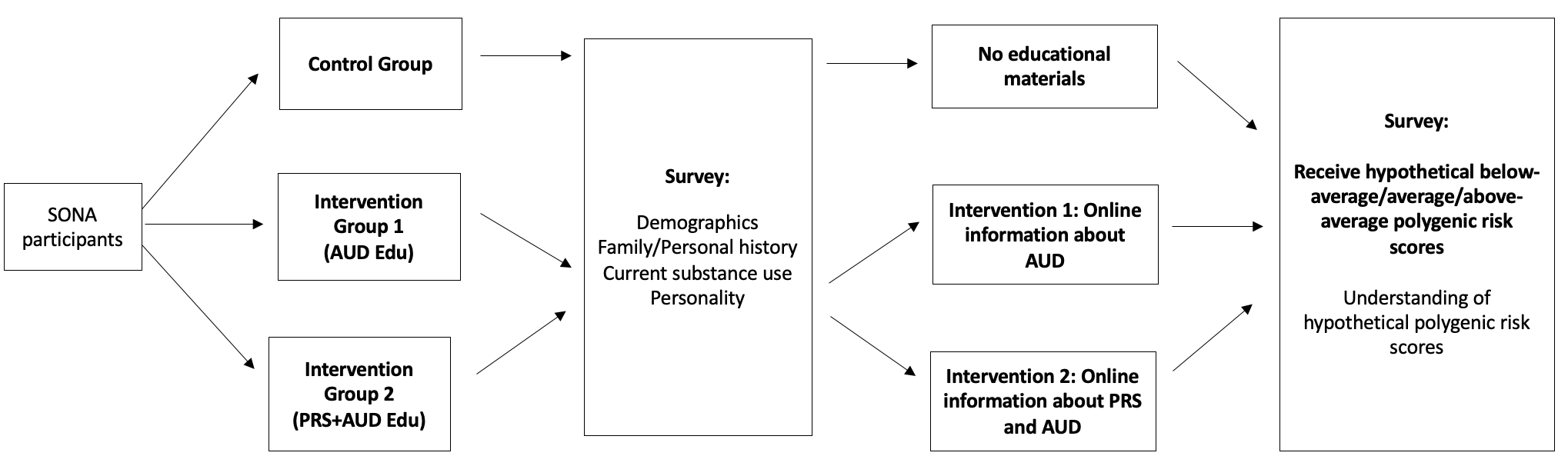


**Supplementary Figure 1.** Flow chart of the randomized controlled trial.


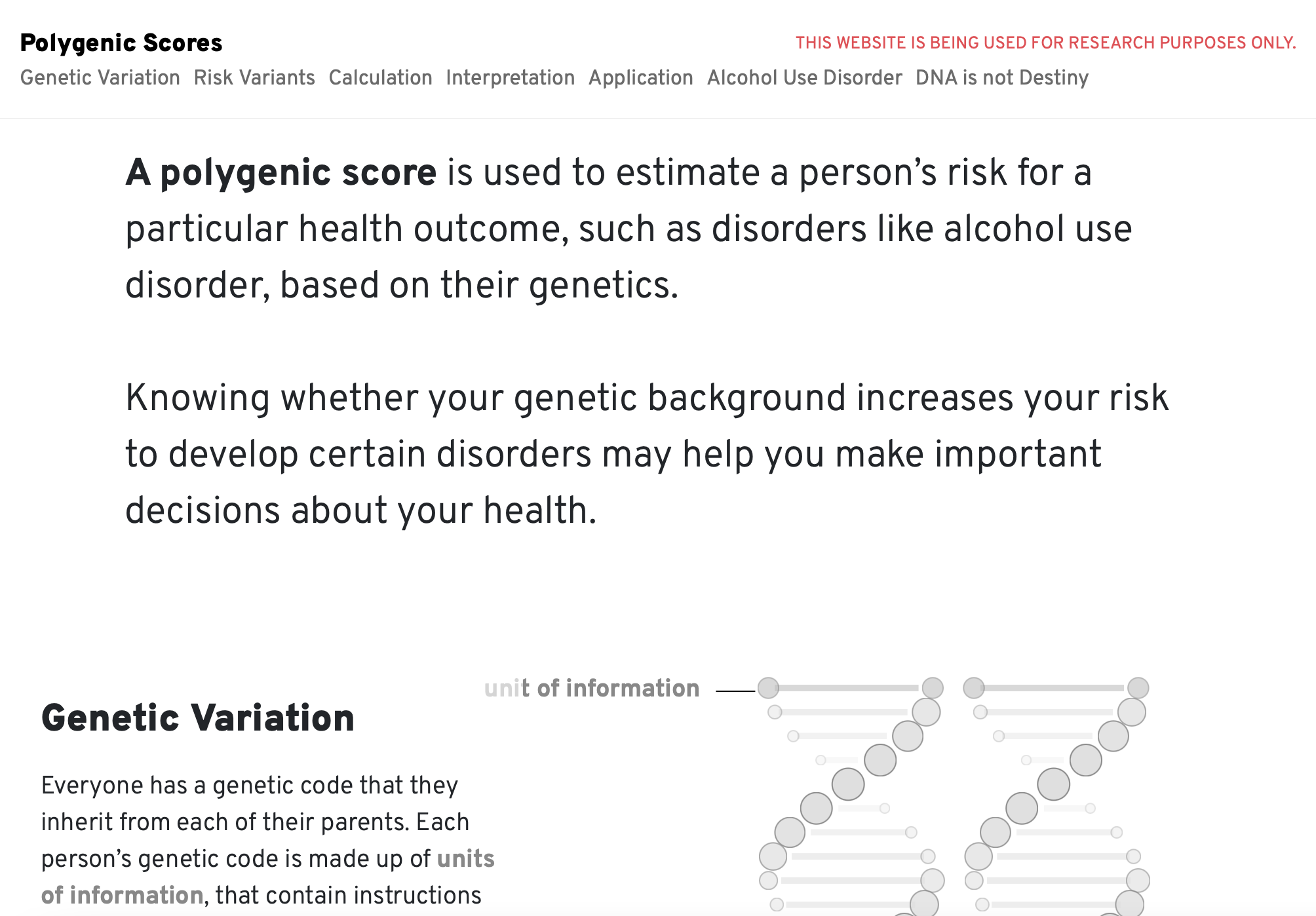


**Supplementary Figure 2.** Image from the educational tool demonstrating the simple text, short sentences, and repetitive phrases that were used throughout the site to enhance comprehension.


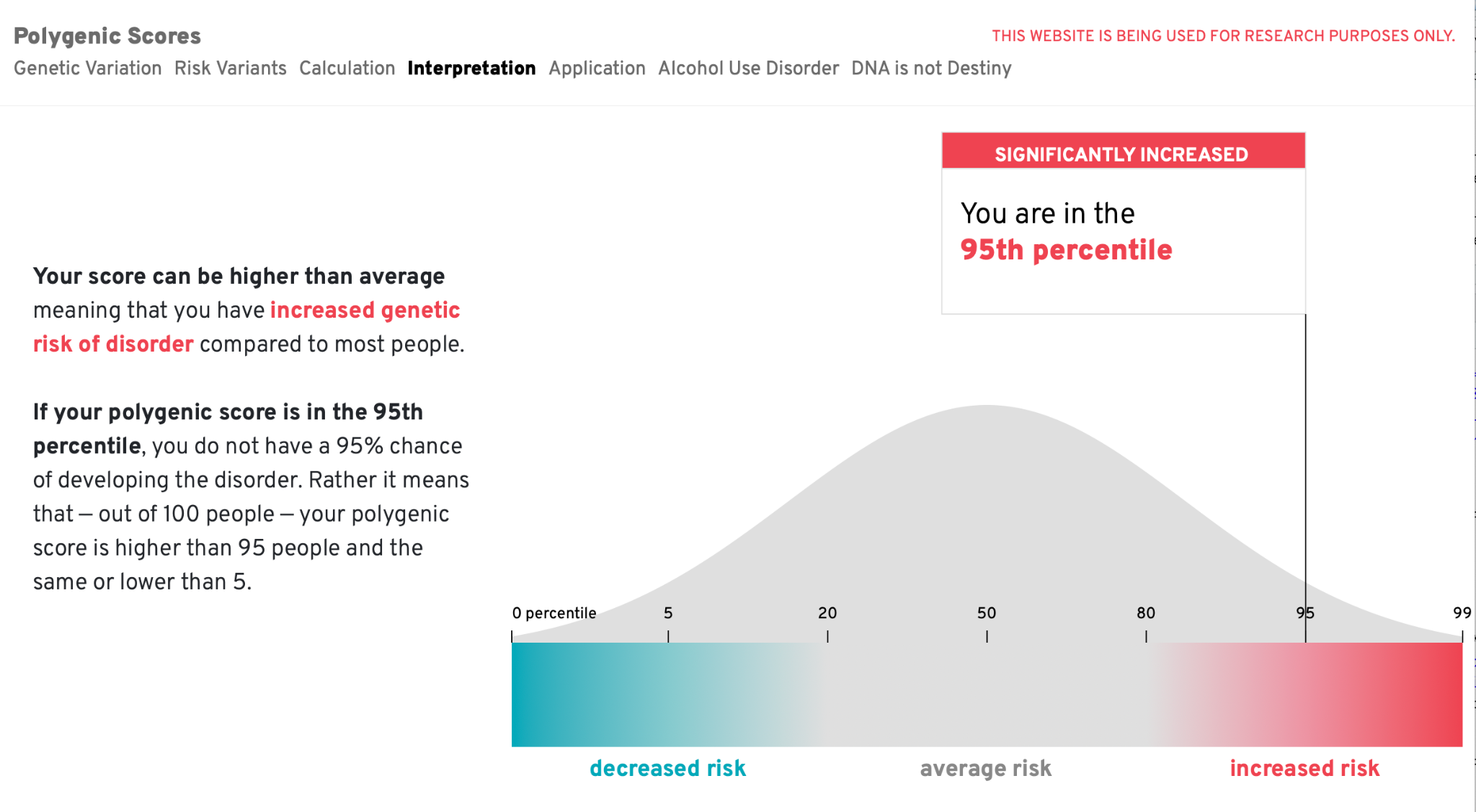


**Supplementary Figure 3.** Image from the educational tool demonstrating how color coding was used to enhance understanding with teal indicating lower genetic risk, gray indicating average genetic risk, and red indicating higher genetic risk.


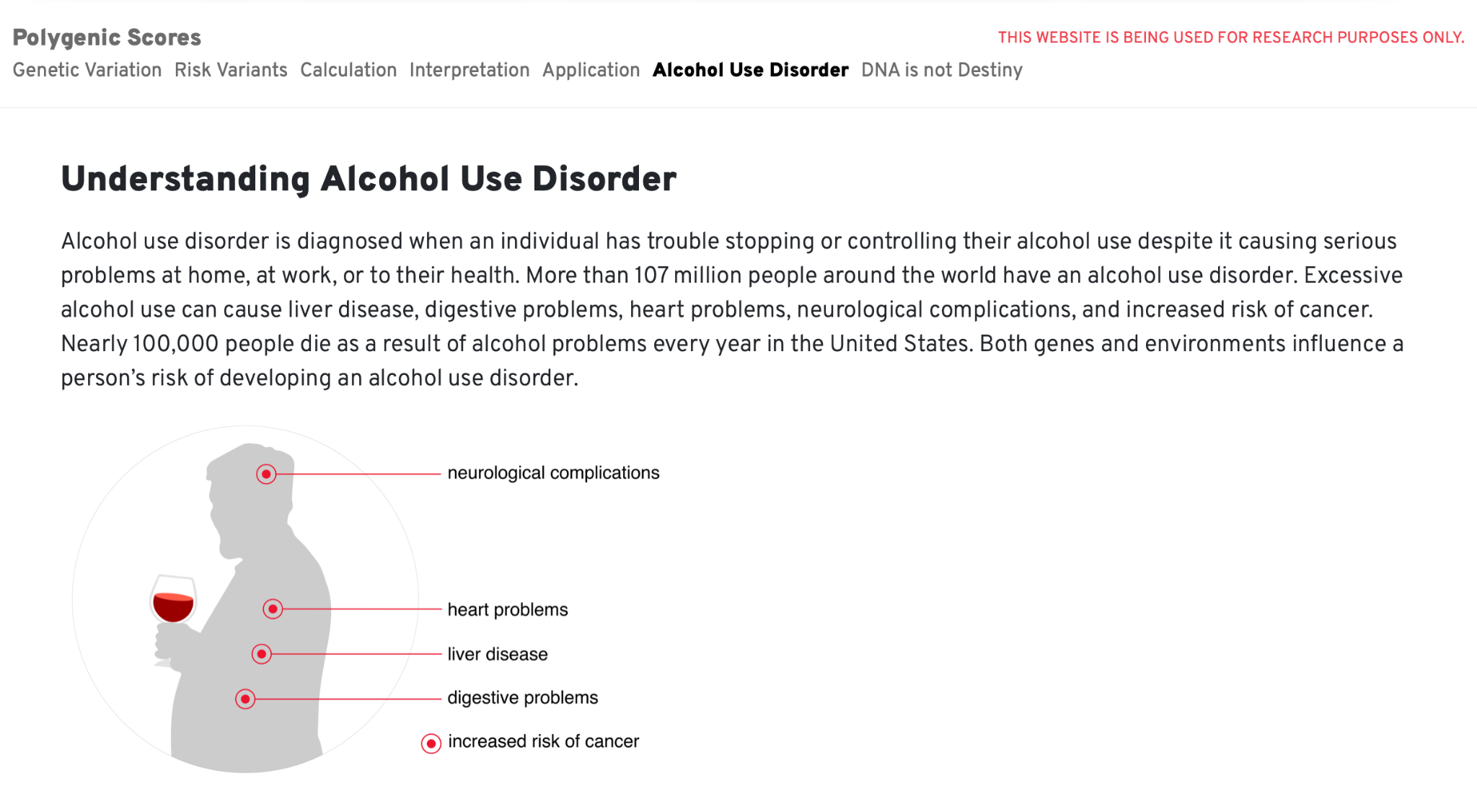


**Supplementary Figure 4.** Image from the educational tool showing the information about alcohol use disorder provided to participants.


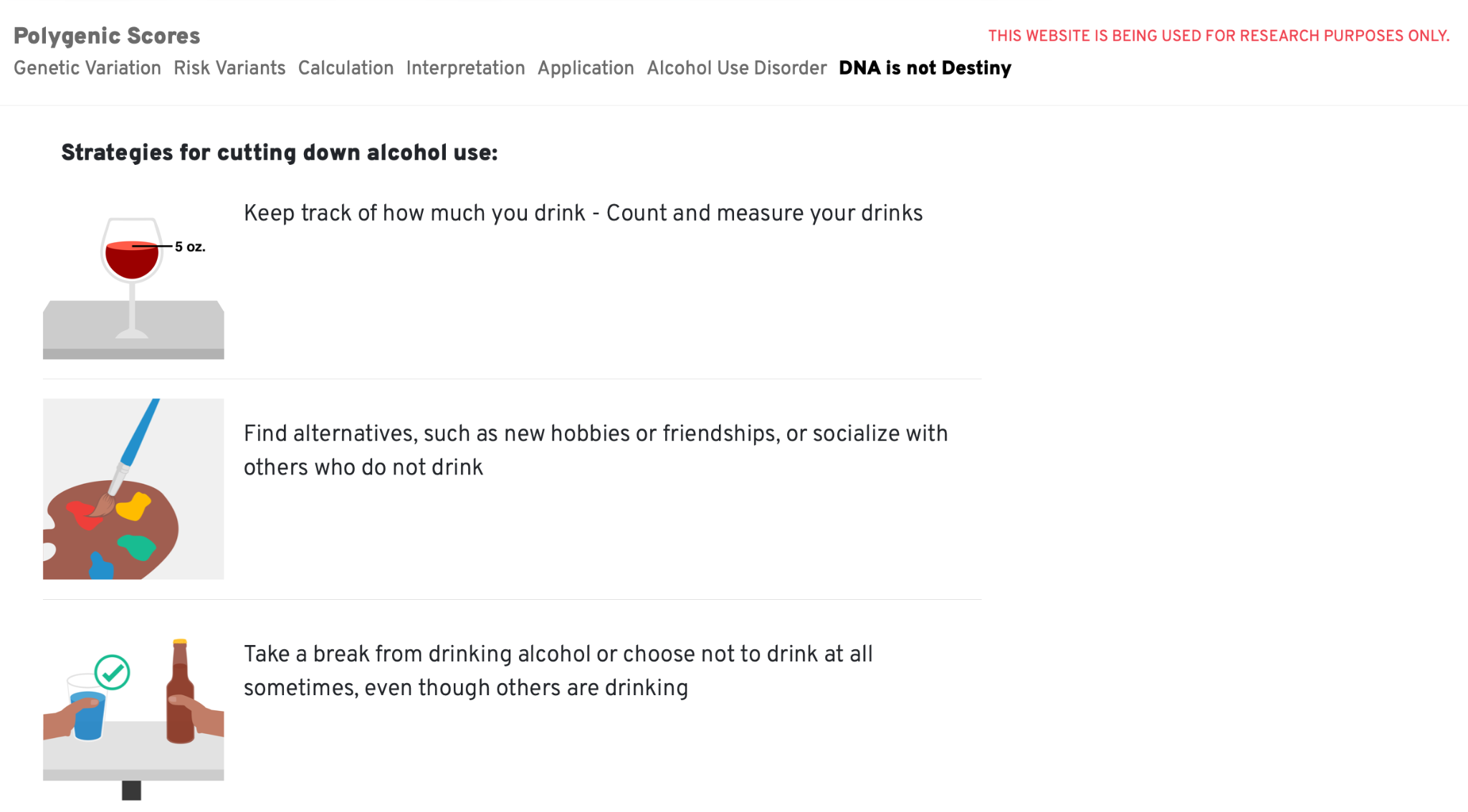


**Supplementary Figure 5.** Image from the educational tool demonstrating the different risk reducing strategies presented to participants using a series of simple graphics and short text.


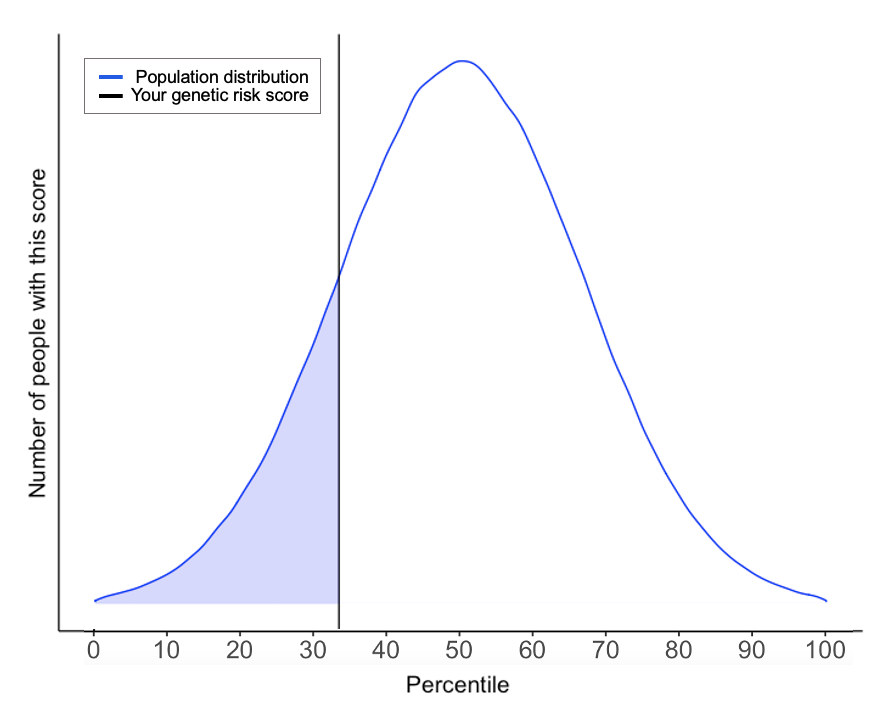

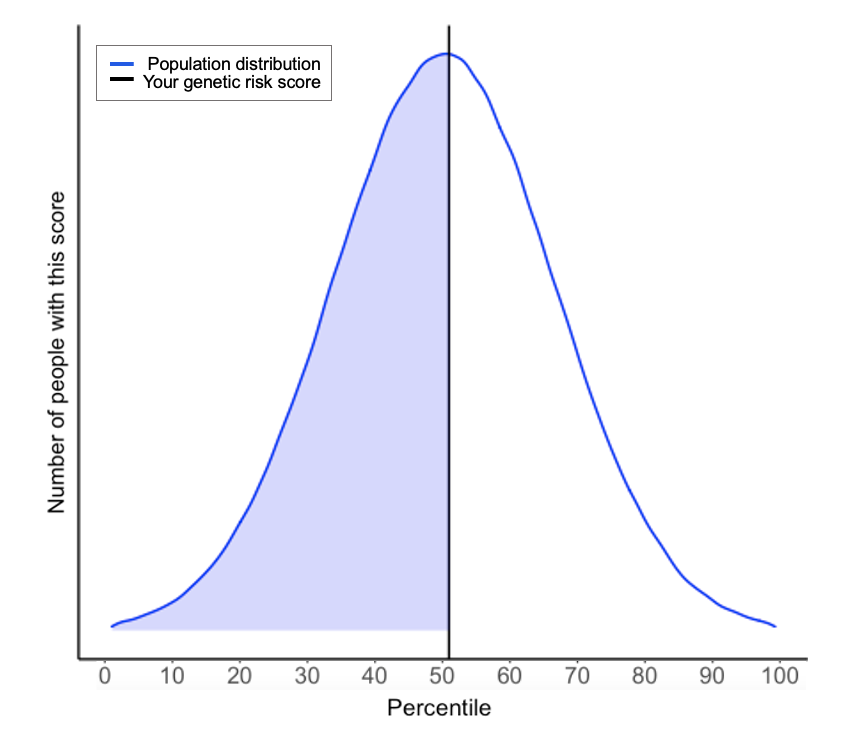

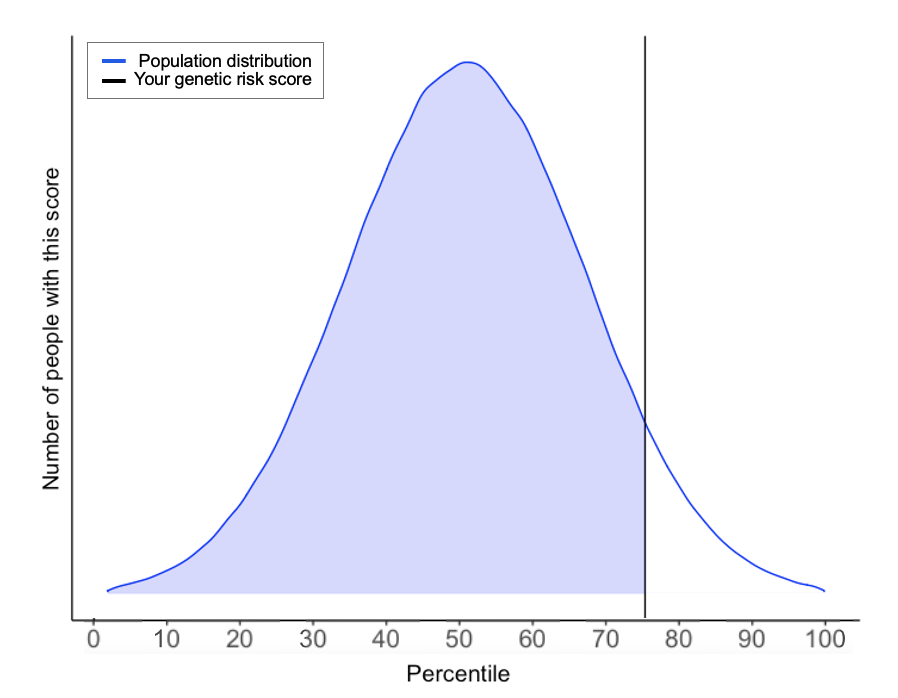


**Supplementary Figure 6.** Graphs that were used to present hypothetical polygenic risk information to the participants.

## Supplementary Tables

**Supplementary Table 1.** Results from the linear regression analysis used to assess robustness of the effect of the intervention while controlling for individual differences.

|  | **Overall Understanding of PRS** | | | |
| --- | --- | --- | --- | --- |
| Variable | b | Std Error | β | p value |
| Sex | -0.417 | 0.383 | -0.065 | 0.277 |
| Age | -0.227 | 0.123 | -0.103 | 0.065 |
| Race/ethnicity | -1.114 | 0.339 | -0.189 | 0.001 |
| Family history | -0.196 | 0.326 | -0.034 | 0.547 |
| Drinking status | 0.229 | 0.363 | 0.037 | 0.530 |
| Extraversion | 0.059 | 0.162 | 0.022 | 0.716 |
| Neuroticism | -0.099 | 0.182 | -0.033 | 0.585 |
| Sensation seeking | -0.558 | 0.258 | -0.135 | 0.031 |
| AUD Edu | 0.794 | 0.390 | 0.129 | 0.043 |
| PRS Edu | 1.154 | 0.390 | 0.187 | 0.003 |
| *Note:* Sex is coded as 0 = male, 1 = female. Race/ethnicity coded as 0 = White, 1 = Non-White. Family history is coded as 0 = no family history of alcohol problems, 1 = family history of alcohol problems in at least one first- or second-degree relative. Drinking status is coded as 0 = does not consume alcohol, 1 = consumed alcohol at least once. AUD Edu is coded as 0 for participants who did not receive educational information about alcohol use disorder and 1 for participants who received information about alcohol use disorder. PRS Edu is coded as 0 for participants who did not receive educational information about polygenic risk scores and 1 for participants who received information about polygenic risk scores. PRS = polygenic risk scores. | | | | |
